# Brain Transcriptomic Changes Associated With the Socially Regulated Behavioural Specialisation of Ant Queens

**DOI:** 10.1111/mec.70614

**Published:** 2026-11-01

**Authors:** Hugo E. Le Lay, Maximilian F. Bolder, Thomas J. Colgan, Romain Libbrecht

**Affiliations:** ^1^ Insect Biology Research Institute, UMR 7261, CNRS University of Tours Tours France; ^2^ Institute of Organismic and Molecular Evolution Johannes Gutenberg University Mainz Germany

**Keywords:** colony foundation, division of labour, gene expression, social behaviour, social insects

## Abstract

Insect societies are characterised by a reproductive division of labour between queens that monopolise egg production and workers that perform all non‐reproductive tasks. The queen specialisation in egg laying is typically considered to be constitutive and independent of environmental conditions, but this common assumption was recently challenged by the finding that the presence of workers both induces and maintains the queen specialisation in several species of ants. However, the molecular changes that translate worker presence into queen behavioural specialisation remain unknown. To investigate the transcriptomic basis of the socially regulated specialisation of ant queens, we produced 93 individual brain transcriptomes from queens of the black garden ant *Lasius niger* that were experimentally maintained in different social environments. Our transcriptomic analyses revealed that 123 genes were differentially expressed between the brains of queens maintained with and without workers. These genes were enriched for processes related to brain energy metabolism and neuronal plasticity and signalling. By investigating the response of selected genes of interest to further manipulations of the social environment, we identified four candidate genes that fully mirrored the queen behavioural changes. Specifically, their expression changes in response to worker presence were reversible and they did not respond to mere cues of the worker presence. These candidate genes included the transcription factor *Krüppel‐homologue 1* and other genes in the juvenile hormone and insulin signalling pathways, both known to regulate multiple aspects of division of labour in social insects. By producing a robust, well‐replicated and well‐controlled investigation of the transcriptomic basis of the behavioural specialisation of ant queens, this study provides a better understanding of the emergence and maintenance of reproductive division of labour in insect societies.

## 1 Introduction

Social insect colonies are characterised by a reproductive division of labour between queens that specialise in egg laying and workers that fulfil non‐reproductive tasks, such as caring for the brood, foraging for food or cleaning the nest (Andersson 1984; Lin and Michener 1972; Robinson 2009). Queens and workers have distinct phenotypes, as their morphology (Divieso et al. 2020; Miyazaki et al. 2005), physiology (Barchuk et al. 2007; Kramer and Schaible 2013) and gene expression (Friedman and Gordon 2016; Gräff et al. 2007; Lockett et al. 2016; Xu and Colgan 2025) underlie their behavioural specialisation. Most studies of reproductive division of labour have focused on the process of caste determination that occurs during larval development (Beetsma 1979; de Azevedo and Hartfelder 2008; Reginato and Cruz‐Landim 2003; Villalta et al. 2016), thus assuming that queen specialisation in egg laying is fixed at the adult stage.

However, in most social insect species, the queens are not yet specialised in egg production at the beginning of their adult life (Majidifar et al. 2024). Young, newly mated queens that found their colony independently (i.e., without the help of workers) express a wide behavioural repertoire to produce the first workers, including the performance of non‐reproductive tasks such as brood care (Augustin et al. 2011; Cassill 2002; Chouvenc 2022; Majidifar et al. 2024; Woodard et al. 2013). Only upon the emergence of the first workers do queens specialise in egg laying, transitioning from a pluripotent to a specialised state (Augustin et al. 2011; Cassill 2002; Majidifar et al. 2024; Woodard et al. 2013).

Experimental studies have revealed that the presence of workers is sufficient and necessary to induce queen specialisation in egg production, but also to maintain it in established colonies (Majidifar et al. 2024; Woodard et al. 2013). Experimental removal of workers causes queens to revert to expressing brood care behaviour, thus resembling founding queens (Majidifar et al. 2024; Woodard et al. 2013). Such a flexible, socially controlled specialisation stands in contrast with the commonly accepted constitutive specialisation of the queen caste. It also differs from other social insect species, such as the termite *Coptotermes gestroi* , in which reproductive individuals irreversibly lose the ability to ensure proper offspring development after colony foundation (Chouvenc 2022; Chouvenc and Su 2017).

Further investigations of the effect of workers on queen specialisation in the ant *Lasius niger* have revealed that queens exposed to the mere presence of worker cues do not become specialised in egg production (Majidifar et al. 2024). For example, founding queens continue to express brood care in the presence of workers that are separated from the queen and brood by a wire mesh, indicating that close contacts between workers and queen, and/or between workers and brood are required to produce queen specialisation (Majidifar et al. 2024). Although the behavioural response of ant queens to various manipulations of the social environment has been previously characterised (Majidifar et al. 2024), the molecular mechanisms that translate the presence of workers into queen behavioural specialisation remain to be investigated.

Several lines of evidence indicate that the effect of workers on the specialisation of ant queens is likely mediated by changes in brain gene expression. First, the social environment influences brain gene expression in queens of other social insect species, as evidenced by the finding that the presence of workers is associated with brain gene expression changes in queens of the bumblebee *Bombus terrestris* (Woodard et al. 2013). In addition, the presence of other queens affects brain gene expression in queens of the ant *Temnothorax rugatulus* (Stoldt et al. 2023). Second, the experimental removal of queens drives profound behavioural and physiological modifications in workers (dos Santos Conceição Lopes et al. 2020; Scharf et al. 2021; Huang et al. 2025; Kohlmeier et al. 2017; Majoe et al. 2021; Negroni et al. 2021), which are associated with brain transcriptomic changes (Huang et al. 2025; Negroni et al. 2021; Peng et al. 2024; Scharf et al. 2021). For example, in the ant *Harpegnathos saltator* , queen loss induces the transition of workers into reproductive gamergates via modifications of the brain transcriptome (Gospocic et al. 2021; Opachaloemphan et al. 2021). Third, removing groups of workers that are specialised in performing specific tasks causes other workers to change their behaviour and adjust their brain gene expression (Fussnecker and Grozinger 2008; Kohlmeier et al. 2018, 2019; Manfredini et al. 2014). Fourth, worker behaviour, physiology and brain gene expression change in response to experimental manipulations of the presence of brood in the ants *Ooceraea biroi* (Libbrecht et al. 2018, 2016; Chandra et al. 2018; Ulrich et al. 2016) and *S. invicta* (Hawkings and Tamborindeguy 2024), as well as in the honey bee *Apis mellifera* (Eyer et al. 2017; Ma et al. 2019). Finally, the presence of social parasites affects the brain gene expression of workers in multiple species of ants (Stoldt et al. 2023; Kaur et al. 2019).

We hypothesised that the induction of queen behavioural specialisation by the presence of workers could involve genes and pathways that respond to changes in the social environment in other contexts and species. Multiple studies have previously reported effects of the social conditions on the expression in the brain of genes associated with the interconnected juvenile hormone (JH) (Eyer et al. 2017; Gospocic et al. 2021; Opachaloemphan et al. 2021), insulin/insulin‐like signalling (Chandra et al. 2018; Kohlmeier et al. 2019; Libbrecht et al. 2018) and vitellogenin pathways (Eyer et al. 2017; Hawkings and Tamborindeguy 2024; Kohlmeier et al. 2019, 2018; Lenhart et al. 2024; Santos et al. 2022; Harrison et al. 2015). Of particular interest is the gene *Krüppel‐homologue 1* (*Kr‐h1*), coding for a JH‐responsive transcription factor, which responds to queen pheromones in honey bee workers (Fussnecker and Grozinger 2008; Shpigler et al. 2010) and drives the reproductive activation and associated behavioural changes in *H. saltator* workers (Gospocic et al. 2021; Opachaloemphan et al. 2021). Also of interest are genes coding for insulin‐like peptides, such as *insulin‐like peptide 2* (*ilp2*), which is differentially expressed between the brains of established, reproductive queens and non‐reproductive workers in many species of ants (Qiu et al. 2018; Chandra et al. 2018), responds to variation in the social environment (Libbrecht et al. 2018, 2016) and regulates reproductive activity in *O. biroi* (Chandra et al. 2018).

To investigate the transcriptomic changes that translate the presence of workers into the behavioural specialisation of ant queens, we conducted a differential expression analysis between the brains of *L. niger* founding queens of similar age that were experimentally maintained with or without workers (Majidifar et al. 2024). We identified a set of genes of interest to be further investigated. We predicted that, if the genes of interest translated modifications of the social environment into behavioural specialisation, the changes in expression in response to further manipulations of the presence of workers and/or larvae should mirror the behavioural responses to these manipulations (Majidifar et al. 2024). These analyses produced a robust set of candidate genes and pathways for the molecular regulation of queen specialisation in ants.

## 2 Methods

To investigate the transcriptomic response of *L. niger* founding queens to experimental manipulations of the presence of workers and/or larvae, we used samples that were collected after behavioural analyses reported in Majidifar et al. (2024) and samples that were specifically produced for this study. All experiments were conducted using *L. niger* queens that were collected in Mainz (Germany) after nuptial flights in July 2019 and July 2020, and each cohort of queens was then kept in a climate chamber at 21°C and 80% humidity in the dark.

### 2.1 Sample Collection

#### 2.1.1 Effect of the Experimental Addition and Removal of Workers

To investigate the effect of experimentally adding and/or removing workers on queen brain gene expression, we used queens from Experiment 2.2.4 in Majidifar et al. (2024). In this experiment, founding queens that had never produced workers were separated into three treatments: (i) queens kept with five larvae and no workers; (ii) queens kept with five larvae and five workers and (iii) queens kept with five larvae that formerly had had workers for three days. The behavioural analyses revealed that queens that had their workers removed performed more brood care than queens with workers, but as much brood care as queens without workers. More details on the experimental methods, statistical analyses and results can be found in Majidifar et al. (2024). Founding queens were maintained in these experimental conditions for 48 h before they were flash‐frozen in liquid nitrogen and stored at −80°C until RNA extraction.

#### 2.1.2 Effect of the Experimental Addition of Workers and Separated Workers

To investigate the effect on queen brain gene expression of experimentally adding workers together with the queen or separated from the queen by a wire mesh, we used queens from Experiment 2.2.12 in Majidifar et al. (2024). In this experiment, founding queens that had never produced workers were separated in three treatments: (i) queens kept with five larvae and no workers; (ii) queens kept with five larvae and three workers in the same arena and (iii) queens kept with five larvae and three workers that were separated from the queens and the larvae by a wire mesh. The behavioural analyses revealed that, contrary to queens kept in the same arena as workers, queens provided with separated workers did not show a reduction in brood care behaviour compared to queens kept without workers. More details on the experimental methods, statistical analyses and results can be found in Majidifar et al. (2024). Founding queens were maintained in these experimental conditions for 24 h before they were flash‐frozen in liquid nitrogen and stored at −80°C until RNA extraction.

To verify that the wire mesh enabled antennal contacts between the queens and the separated workers, we reanalyzed the 1 h‐long videos that were recorded 2, 6 and 24 h after the experimental set‐up (Majidifar et al. 2024). For each queen, we used focus animal sampling (Altmann 1974) with the BORIS software (version 8.0.5) (Friard and Gamba 2016) to measure the number of times that queens passed their antennae through the wire mesh to make antennal contacts with the separated workers, the duration of these antennal contacts, and the number of times the queens passed their antennae through or above the wire mesh without touching workers.

#### 2.1.3 Effect of the Experimental Manipulation of the Presence of Larvae

To study the effect on queen brain gene expression of expressing brood care and being in contact with larvae, we used *L. niger* founding queens that were collected after the nuptial flight in Mainz in July 2020. The experiment started 70 days after collecting. The founding queens, which had not produced workers yet, were distributed in two treatments: (i) queens kept with six larvae and (ii) queens kept without larvae. The larvae were sourced from field‐collected colonies that also served as sources of workers in Majidifar et al. (2024). During the experiment, the queens were kept in the same conditions as in Experiment 2.2.4 in Majidifar et al. (2024). After 72 h, the queens were flash‐frozen in liquid nitrogen and stored at −80°C until RNA extraction.

### 2.2 Brain Dissection, RNA Extraction and Sequencing

#### 2.2.1 Sample Selection

For the experiments involving manipulations of the presence of workers, we selected samples for RNA sequencing (RNA‐seq) based on the brood care data from Majidifar et al. (2024). For each treatment, we selected three queens from each quartile of brood care level (i.e., the number of scans where brood care was detected), yielding a total of 12 representative samples per treatment. In the treatment where queens were separated from workers by a wire mesh, we removed three samples because the video analyses revealed that workers managed to enter the arena of the queen. For the experiment that manipulated the presence of larvae, we randomly selected 12 queens per treatment. Thus, the selection process resulted in a total of 93 selected samples (Table 1).

**TABLE 1:** Sample selection for RNA‐seq analyses.

Name and description of the experiments	Experiment A: Experimental addition and removal of workers	Experiment B: Experimental addition of workers and separated workers	Experiment C: Experimental manipulation of the presence of larvae
Experiment number in Majidifar et al. (2024)	Experiment 2.2.4	Experiment 2.2.12	Not from Majidifar et al. (2024)
Number of RNA‐seq samples	Queens without workers (12 samples) Queens with workers (12 samples) Queens with removed workers (12 samples)	Queens without workers (12 samples) Queens with workers (12 samples) Queens with separated workers (9 samples)	Queens without larvae (12 samples) Queens with larvae (12 samples)

*Note:* The table includes information on the origin of samples in Majidifar et al. (2024) and the number of RNA‐seq samples for each experimental manipulation of the social environment.

#### 2.2.2 Brain Dissection

Brain dissections were performed over several days and the order in which samples were dissected was randomised across experiments. To dissect the brain, we first removed the queen from the cryotube and transferred it to a silicon slip, set in ice, under a stereomicroscope (Leica S9i; Leica Microsystems, Germany). To manipulate the queen, we used two micro‐forceps (No. 11295‐10; F.S.T., Canada). We first removed the head, placed it in a cooled 1× PBS droplet and removed the antennae at the base. The rest of the queen body and the antennae were returned to the cryotube and stored in a box filled with dry ice. We opened the head capsule by cutting dorsally from the clypeus along the frontal line up to the hind margin of the head capsule using a micro‐scissor (No 15000‐08; F.S.T., Canada). This was followed by two cuts, 90° from the first, resulting in a T‐shaped cut. Afterwards, we removed the cuticle encompassing the cut using the micro‐forceps. To sever the connection to the retina, we used one micro‐forceps to scrape along the inside of the compound eyes. Any additional cuticle obscuring the brain above the ocelli and hind margin of the head capsule was removed. The brain, once visible, was carefully lifted out of the head capsule by grabbing it from below and placed in another cooled 1× PBS droplet. We then transferred the brain to a pre‐chilled vial containing 100 μL of TRIzol and kept on ice. After dissection, all samples were transferred and stored at −80°C.

#### 2.2.3 RNA Extraction and Sequencing

From each sample, the total RNA was extracted using a TRIzol:chloroform extraction protocol followed by purification using a Qiagen RNeasy extraction kit with a modified version of its protocol, in which the ethanol used for the initial step was changed from 70% to 100%, and the volumes of RPE buffer used in the first and second wash steps were adjusted to 400 and 300 μL, respectively. Total RNA was shipped on dry ice to a commercial supplier (Novogene, UK), where quality assessment was performed using an Agilent Bioanalyzer before an individual mRNA‐enriched library preparation was performed for each sample. The libraries were individually barcoded, pooled and sequenced on an Illumina Plus X sequencing platform. The sequencing generated a minimum of 8 Gb per library, corresponding to 26 million paired‐end 150 bp (PE150) reads per sample.

### 2.3 Gene Expression Analyses

#### 2.3.1 From Raw Sequencing Files to Read Count Tables

Raw paired‐end reads were trimmed and filtered using *Fastp* v.0.20.1 (Chen et al. 2018) to remove adapters and reads with more than 15 ambiguous bases and/or shorter than 120 bp. Quality assessment of raw and filtered reads was performed via *FastQC* v.0.12.1 (Andrew 2017) with output files summarised and visualised using *MultiQC* v.129 (Ewels et al. 2016). Abundance of transcripts per sample was quantified using *Kallisto* v.0.50 (Bray et al. 2016) by pseudo‐aligning filtered RNA‐seq data against the *L. niger* annotated coding regions (GAGA‐0177). The genome and associated annotation files were downloaded from the electronic research data archive of the University of Copenhagen (https://erda.ku.dk/archives/a3a1fd0ea7f773de242c9a0405bfa57b/published‐archive.html) (Masson et al. 2024; Vizueta et al. 2025). The resulting 93 abundance files were loaded into R using *tximport* v.1.37.2 (Soneson et al. 2016), summarised to gene‐level counts before being analysed using the R package *DESeq2* (Love et al. 2014). To filter out lowly expressed genes, we retained only those genes that exhibited non‐zero expression in at least 25 samples. Of the 13,903 genes annotated in the *L. niger* genome, 11,269 genes met this criterion and were retained for downstream analysis using R (v.4.4.2) and RStudio (v.2024.04.2). Code and input data for all analyses are available in Data S1.

#### 2.3.2 Analysis of the Effect of Worker Presence on Queen Brain Gene Expression

To produce a conservative and robust analysis of the transcriptomic response of founding queens to the experimental manipulation of the presence of workers, we combined brain RNA‐seq samples from two different experiments that involved the same control treatments: queens kept without workers (negative control, *n* = 24); and queens experimentally provided with workers (positive control, *n* = 24). We used 24 queens from Experiment 2.2.4 (12 without workers; 12 with workers) and 24 queens from Experiment 2.2.12 (12 without workers; 12 with workers) in Majidifar et al. (2024) (Table 1). To implement the differential expression analysis while controlling for batch effects driven by differences between the two experiments that produced the samples (e.g., number of workers, timing of sample collection, year of the experiment), we performed a likelihood‐ratio test (LRT) using the *DESeq2* package (Love et al. 2014) to compare a full model that included worker presence and batch (i.e., experiment of origin) as explanatory variables to a reduced model that only included batch (Love et al. 2014; Qian and Marianthi 2016). Importantly, the two experiments contributed the same number of samples to each treatment, thus the experiment of origin of the samples was not confounded with the presence or absence of workers in the analysis. *p* values were adjusted for multiple testing using the default Benjamini and Hochberg method (Love et al. 2014).

To investigate the biological functions of the differentially expressed genes, we conducted a Gene Ontology (GO) term enrichment analysis. We identified *biological process* and *molecular function* GO terms that were overrepresented in the list of differentially expressed genes using the *L. niger* GO annotation file (Masson et al. 2024; Vizueta et al. 2025). We used the *topGO* package (Alexa and Rahnenführer 2017) with the weight01 algorithm, Fisher's exact test and default node size to identify enriched GO terms (*p* < 0.01) associated with at least three genes in the list of differentially expressed genes.

#### 2.3.3 Identification of Genes of Interest

We manually inspected the functional annotations of the differentially expressed genes using the *L. niger* genome annotation (Masson et al. 2024; Vizueta et al. 2025) to identify genes of interest for the regulation of queen behavioural specialisation in response to changes in the social environment. We defined genes of interest as those with reported behavioural functions associated with their expression in the brain of insects.

#### 2.3.4 Analysis of the Effect of Worker Removal on the Expression of Genes of Interest (Experiment A)

We investigated whether changes in the expression of genes of interest in the brain of founding queens in response to the experimental removal of workers mirrored the behavioural response (Majidifar et al. 2024). To do so, we used 36 queens from Experiment 2.2.4 in Majidifar et al. (2024) (Table 1) to compare the expression of the genes of interest among treatments: queens without workers (*n* = 12); queens with workers (*n* = 12) and queens with workers removed (*n* = 12). We extracted the normalised counts for the genes of interest using the *counts()* function of the *DESeq2* package (Love et al. 2014), and conducted ANOVAs using the *car* package (Fox and Weisberg 2018) on linear models that explained gene counts (log‐transformed when necessary) by the treatment. We verified that the assumptions for linear models were met using the *DHARMa* package (Hartig 2026). Whenever the analyses detected an effect of the experimental treatment, we used the *emmeans()* function from the *emmeans* package (Lenth 2017) to perform pairwise comparisons. Our criteria to conclude that a gene of interest mirrored the behavioural response was that its expression should be affected by a main effect of treatment, differ between queens with workers and queens with workers removed, and go in the same direction for queens without workers and queens with workers removed when compared to queens with workers (Majidifar et al. 2024).

#### 2.3.5 Analysis of the Effect of Separated Workers on the Expression of Genes of Interest (Experiment B)

We tested whether changes in the expression of genes of interest in response to the presence of workers separated by a wire mesh mirrored the behavioural response (Majidifar et al. 2024). To do so, we used 33 queens from Experiment 2.2.12 in Majidifar et al. (2024) (Table 1) to compare the expression of the genes of interest among treatments: queens without workers (*n* = 12); queens with workers (*n* = 12) and queens with separated workers (*n* = 9). To do so, we applied the same method that we used to test the effect of worker removal. Similarly, our criteria to conclude that a gene of interest mirrored the behavioural response was that its expression should be affected by a main effect of treatment, differ between queens with workers and queens with separated workers, and go in the same direction for queens without workers and queens with separated workers when compared to queens with workers (Majidifar et al. 2024).

#### 2.3.6 Analysis of the Effect of Larvae on the Expression of the Genes of Interest (Experiment C)

We studied the effect of interacting with larvae on the expression of the genes of interest by comparing queens kept with six larvae (*n* = 12) and queens kept without larvae (*n* = 12) (Table 1). To do so, we extracted normalised counts for the genes of interest and performed ANOVAs on linear models explaining the read counts by the presence of larvae.

## 3 Results

To better understand the molecular mechanisms that translate the presence of workers into behavioural specialisation in *L. niger* founding queens, we used 93 brain RNA‐seq samples to investigate the transcriptomic changes associated with a series of experimental modifications of the social environment that produced queen behavioural variation (Majidifar et al. 2024).

### 3.1 Effect of the Presence of Workers on Brain Gene Expression in Queens

The experimental addition of workers was found to drastically reduce the brood care behaviour of founding queens (Majidifar et al. 2024). We hypothesised that the expression of genes that translate changes in the social environment into queen behavioural specialisation would be affected by the experimental addition of workers. We identified 123 genes that differed in expression between founding queens that were either kept without workers or experimentally provided with workers (adjusted *p* < 0.05): 63 genes (51.2%) were upregulated in the brain of queens kept without workers, and 60 genes (48.8%) were upregulated in the brain of queens provided with workers (Figure 1A, Table S1).

**FIGURE 1:**
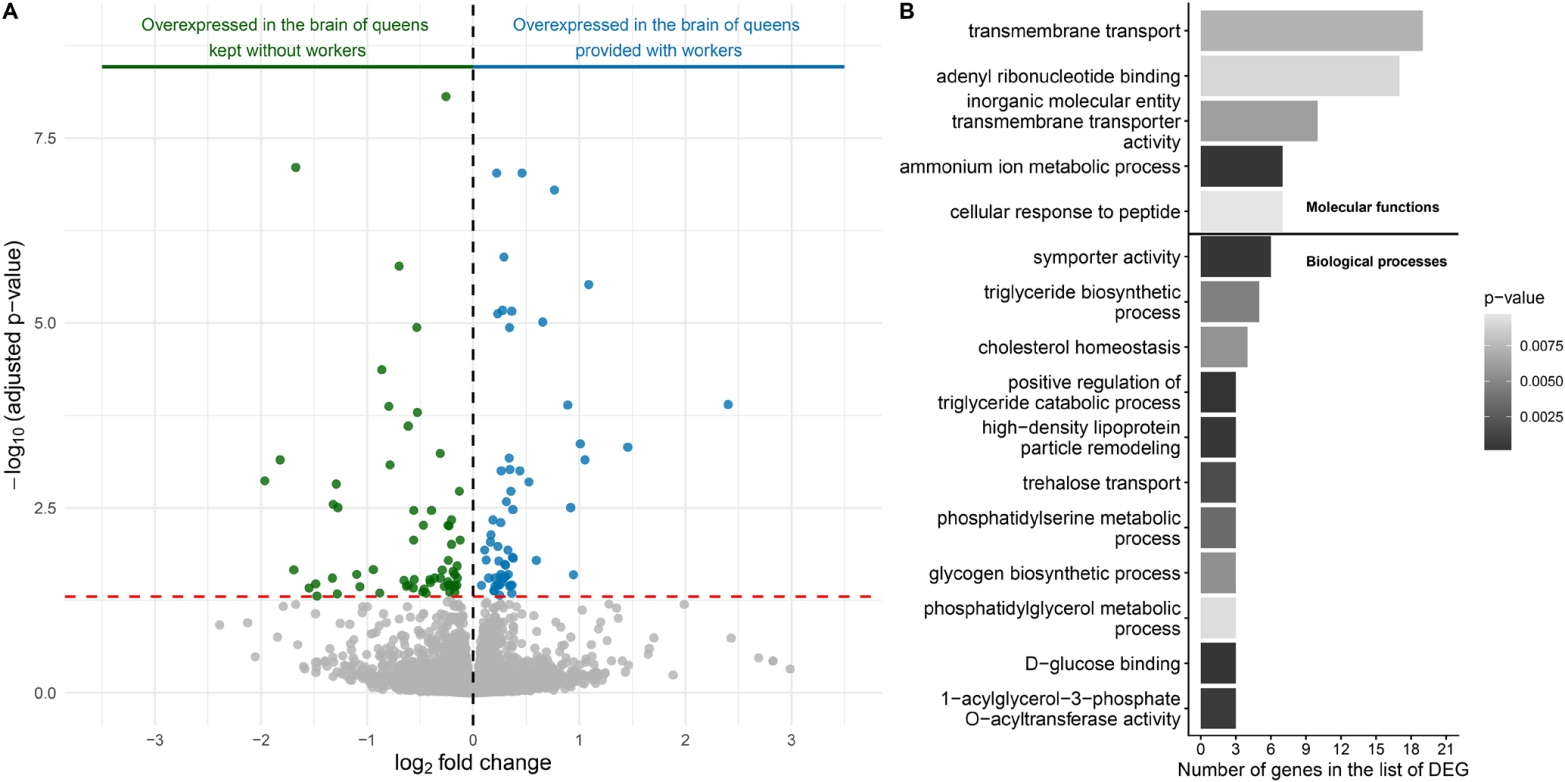
The experimental addition of workers affected the brain gene expression of *Lasius niger* founding queens. (A) Volcano plot summarising the differential expression analysis. Coloured dots represent the 123 genes that were differentially expressed between queens without (*n* = 24) and with (*n* = 24) workers (adjusted *p* < 0.05; above the red dashed line). Green dots are genes that were overexpressed in the brain of queens without workers. Blue dots are genes that were overexpressed in the brain of queens that were experimentally provided with workers. (B) The GO term enrichment analysis revealed that five GO terms for molecular functions and 11 GO terms for biological processes were enriched in the list of differentially expressed genes (DEG).

To assess whether specific functions were overrepresented in the list of differentially expressed genes, we conducted a GO term enrichment analysis. We found 16 GO terms to be enriched (*p* < 0.01): 11 GO terms for biological processes and five GO terms for molecular functions (Figure 1B, Table S2). Five of the enriched GO terms were related to functions and processes associated with brain energy metabolism, such as carbohydrate metabolism and transport (GO:0015771, *trehalose transport*; GO:0005978, *glycogen biosynthetic process*; GO:0005536, *D‐glucose binding*) and lipid metabolism (GO:0010898, *positive regulation of triglyceride catabolic process*; GO:0019432, *triglyceride biosynthetic process*). We also found enrichment of three GO terms involved in membrane remodelling and neuronal plasticity (GO:0034375, *high‐density lipoprotein particle remodelling*; GO:0006658, *phosphatidylserine metabolic process*; GO:0046471, *phosphatidylglycerol metabolic process*). Finally, five other enriched GO terms related to neurotransmission and signalling regulation, such as the transport of ions and metabolites across membranes (GO:0015293, *symporter activity*; GO:0015318, *inorganic molecular entity transmembrane transport*; GO:0055085, *transmembrane transport*; GO:0097164, *ammonium ion metabolic process*) and the response to peptides (GO:1901653; *cellular response to peptide*).

### 3.2 Identification of Genes of Interest

We inspected the list of 123 differentially expressed genes manually to identify genes of particular interest, which we defined as genes expressed in the brain with reported behavioural functions in insects. Using these criteria, we identified 13 genes of interest (Table 2): seven genes (53.8%) were overexpressed in the brain of queens kept without workers, and six genes (46.2%) were overexpressed in the brain of queens kept with workers (Figure 2, Figure S1). We then investigated whether and how genes of interest responded to further manipulations of the presence of workers and larvae (Majidifar et al. 2024).

**TABLE 2:** Reported behavioural functions of the genes of interests.

Gene annotation (abbreviation)	Gene ID	Molecular pathways and behavioural functions
*Krüppel‐homologue 1 (Kr‐h1)*	*Lnig_g01448*	*Kr‐h1* codes for a JH‐responsive transcription factor (He and Zhang 2022) *Kr‐h1* brain expression regulates behavioural specialisation and reproductive activation in bees and ants (Fussnecker and Grozinger 2008; Gospocic et al. 2021; Opachaloemphan et al. 2021; Shpigler et al. 2010)
*3‐phosphoinositide‐dependent protein kinase (PDK1)*	*Lnig_g11740*	*PDK1* codes for a kinase implicated in the insulin signalling pathway (Wu and Brown 2006) *PDK1* brain expression correlates with behavioural variation in bees and *Drosophila* (Haroon et al. 2020; Wang et al. 2009; Wu and Brown 2006)
*Insulin*	*Lnig_g17128*	*Insulin* codes for an insulin‐like peptide (Badisco et al. 2013; Weger and Rittschof 2024) The brain expression of genes coding for insulin‐like peptides correlates with worker behavioural variation in ants and bees, reproductive activity in ants and regulates the social control of behaviour and reproduction in clonal raider ants (Ament et al. 2008; Chandra et al. 2018; Chen et al. 2023; Kohlmeier et al. 2019; Libbrecht et al. 2018; Qiu et al. 2018)
*Juvenile hormone esterase*	*Lnig_g08195*	*Juvenile hormone esterase* and *Esterase E4* code for JH esterase enzymes (Mackert et al. 2008) JH esterase enzymes degrade JH and influence its level in the brain, which affects gene expression (e.g., *vitellogenin* genes) and worker behavioural variation in ants and bees (Amsalem et al. 2014; Brito et al. 2021; Ju et al. 2023; Pandey and Bloch 2015; Starkey and Tamborindeguy 2023; Wheeler et al. 2013)
*Esterase E4*	*Lnig_g15862*	
*Protein doublesex*	*Lnig_g04508*	*Protein doublesex* codes for a protein that drives sex determination (Klein et al. 2016; Rideout et al. 2010) *Protein doublesex* brain expression regulates male‐specific neuron development in flies, and correlates with the expression of reproductive and non‐reproductive behaviours in ants, bees and flies (Johnson and Jasper 2016; Klein et al. 2016; Rideout et al. 2010)
*Protein henna*	*Lnig_g13064*	*Protein henna* codes for a protein involved in the synthesis of dopamine (Li et al. 2025) *Protein henna* brain expression regulates the expression of gregarious behaviours in locusts (Burrows et al. 2011; Li et al. 2025)
*Prohormone‐2*	*Lnig_g06899*	*Prohormone‐2* codes for a prohormone convertase that enzymatically digests precursors of neuropeptides (Han et al. 2015) *Prohormone* genes enable neuropeptide synthesis, which correlates with worker behavioural variation and division of labour in bees (Han et al. 2015)
*Pheromone‐binding protein Gp‐9*	*gLnigOBP1*	*Pheromone‐binding protein Gp‐9* codes for a chemoreceptive pheromone‐binding protein (Opachaloemphan et al. 2021; Zhang et al. 2024) *Pheromone‐binding protein Gp‐9* brain expression regulates the social structure in fire ants, and correlates with worker behavioural and reproductive variation in ants (Opachaloemphan et al. 2021; Zhang et al. 2024)
*Gustatory receptor which mediates acceptance or avoidance behaviour, depending on its substrates (GR81)*	*gLnigGR81*	*GR81* codes for a chemoreceptive gustatory receptor The brain expression of genes coding for gustatory receptors regulates worker behavioural variation in bees (Paerhati et al. 2015; Simcock et al. 2017)
*Facilitated trehalose transporter Tret1* (*Tret1a*)	*Lnig_g01559*	*Tret1* and *Tret1‐2 homologue* code for trehalose transporters (Kanamori et al. 2010; Saleh et al. 2021; Zhang et al. 2024) The brain expression of genes coding for trehalose transporters correlate with worker behavioural variation, foraging behaviour and/or social dominance in ants (Kanamori et al. 2010; Quque et al. 2023; Zhang et al. 2024)
*Facilitated trehalose transporter Tret1* (*Tret1b*)	*Lnig_g11948*	
*Facilitated trehalose transporter Tret1‐2 homologue* (*Tret1‐2 homologue*)	*Lnig_g10144*	

*Note:* Gene annotations and gene ID come from the *Lasius niger* genome annotation (Masson et al. 2024; Vizueta et al. 2025). For some of the genes of interest, we also provide abbreviated gene annotations (in parenthesis).

**FIGURE 2:**
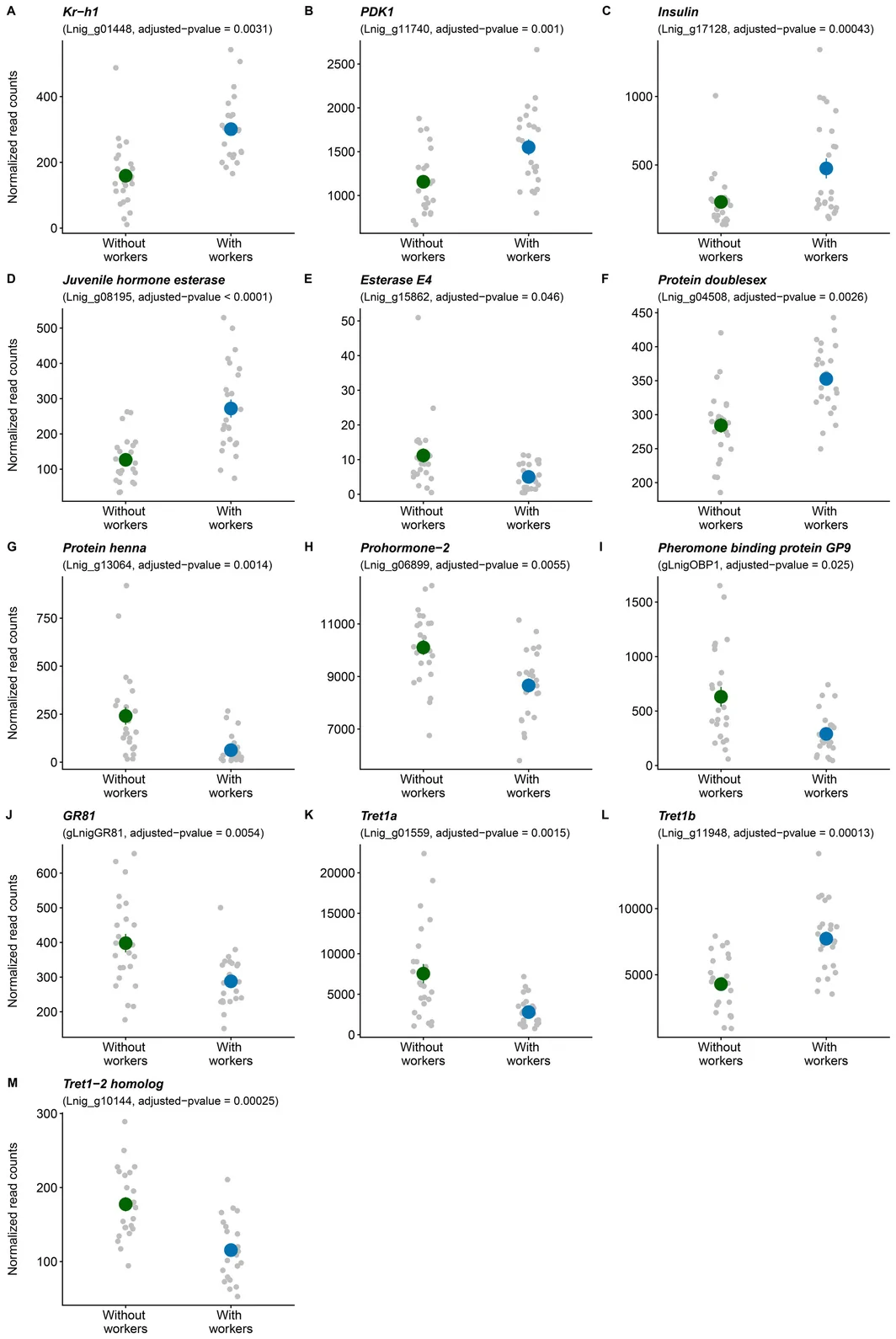
Brain expression patterns of the genes of interest. Coloured points and error bars represent mean ± SE of normalised read counts for queens kept without workers (green, *n* = 24) and queens provided with workers (blue, *n* = 24). Grey dots represent individual data points.

### 3.3 Effect of Worker Removal on the Expression of the Genes of Interest (Experiment A)

First, we studied the effect of the experimental removal of workers on the expression of genes of interest. *L. niger* founding queens of similar age were exposed to one of the following conditions: without workers, experimentally provided with workers, and provided with workers that were then removed after three days. The behavioural analyses revealed that after worker removal, queens expressed more brood care behaviour than queens kept with workers, and similar levels of brood care as queens that never had any workers (Majidifar et al. 2024). These results showed that the behavioural specialisation of *L. niger* queens is reversible. We hypothesised that genes regulating behavioural specialisation would also show reversible changes in expression upon manipulations of the presence of workers.

We argued that for genes of interest to mirror the reversible behavioural response, their expression should be affected by the experimental manipulation of workers (main effect of treatment), differ between queens with workers and queens with workers removed, and go in the same direction for queens without workers and queens with workers removed when compared to queens with workers. We found that six out of the 13 genes of interest showed such reversible expression patterns (Table S3), thus mirroring the behavioural response to worker removal. These genes were *Kr‐h1* (Lnig_g01448), *Juvenile hormone esterase* (Lnig_g08195), *Insulin* (Lnig_g17128), *Pheromone‐binding protein Gp‐9* (gLnigOBP1), *Tret1‐2 homologue* (Lnig_g10144) and *Prohormone‐2* (Lnig_g06899) (Figure 3, Figure S2). The brain expression of the other genes of interest did not differ between queens with workers and queens with workers removed (Figure S2, Table S3), indicating that the expression changes in response to the addition of workers were not reversible, and thus differed from the behavioural response (Majidifar et al. 2024).

**FIGURE 3:**
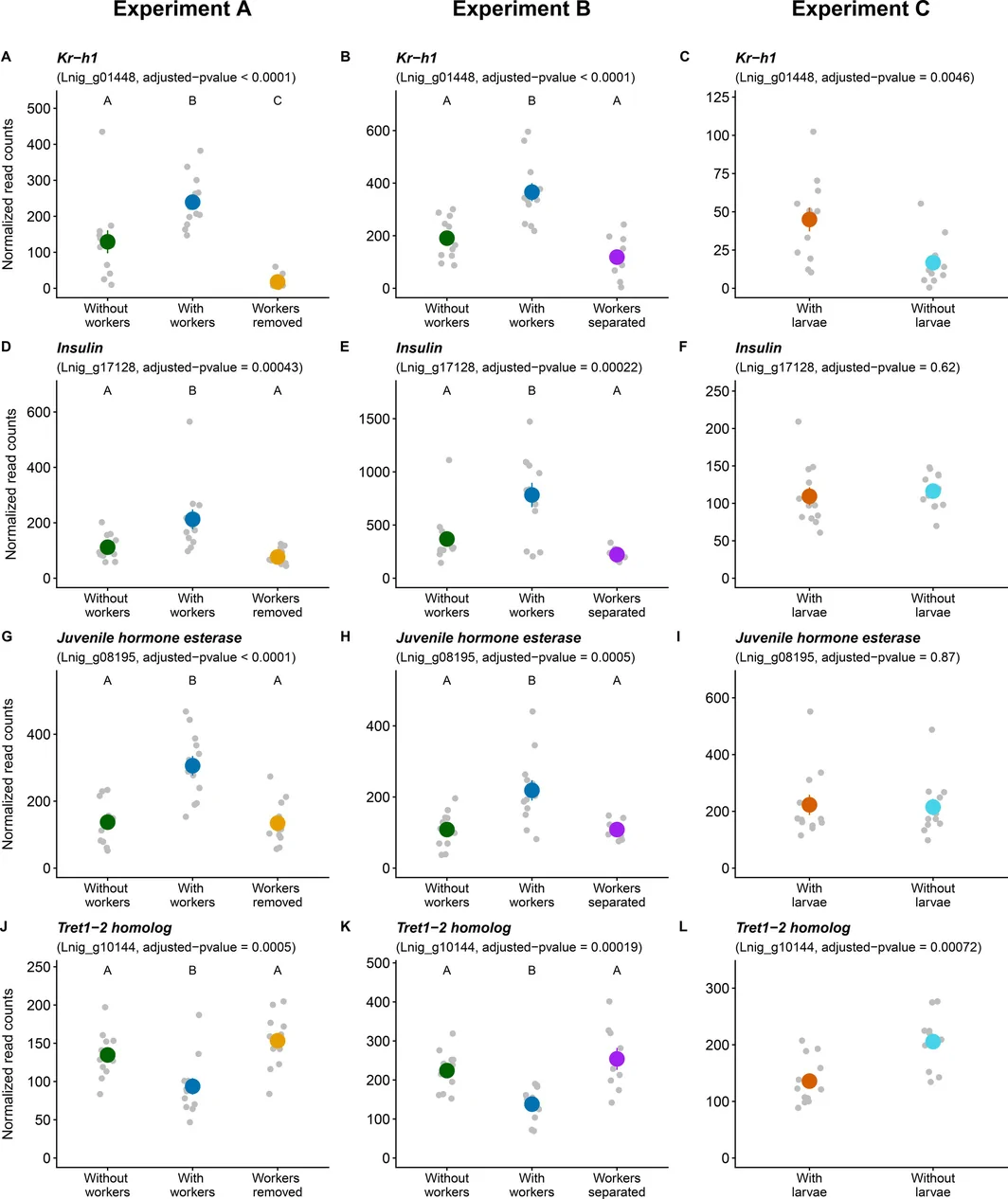
Effect of worker removal (Experiment A), workers separated by a wire mesh (Experiment B) and presence of larvae (Experiment C) on queen brain expression of four genes of interest: *Kr‐h1* (A–C), *Insulin* (D–F), *Juvenile hormone esterase* (G–I) and *Tret1‐2 homologue* (J–L). Coloured points and error bars represent mean ± SE of normalised read counts. Experiment A included queens without workers (green; *n* = 12), queens with workers (blue; *n* = 12) and queens with workers removed (orange; *n* = 12). Experiment B included queens without workers (green; *n* = 12), queens with workers (blue; *n* = 12) and queens with workers separated by wire mesh (purple; *n* = 9). Experiment C included queens without larvae (light blue; *n* = 12) and queens with six larvae (dark orange; *n* = 12). Letters indicate statistically supported differences between groups. Grey dots represent individual data points.

### 3.4 Effect of Separated Workers on the Expression of the Genes of Interest (Experiment B)

Second, we investigated the effect of providing workers that were separated from the queen by a wire mesh. We verified that the set‐up allowed queens to detect the presence of workers via antennal contacts through the wire mesh (Figure S3). We found that, contrary to queens that were placed in the same area as workers, queens provided with separated workers did not respond with changes in their brood care behaviour (Majidifar et al. 2024). We hypothesised that genes regulating the socially driven behavioural specialisation of queens would show changes in expression in the presence of workers, but not in the presence of workers that were separated by a wire mesh.

We argued that if genes of interest did mirror the behavioural response, their expression should be affected by the experimental manipulation of workers (main effect of treatment), differ between queens with workers and queens with separated workers, and go in the same direction for queens without workers and queens with separated workers when compared to queens with workers. We found that nine of the 13 genes of interest showed such expression patterns (Figure 3, Figure S4, Table S4): *Kr‐h1* (Lnig_g01448), *PDK1* (Lnig_g11740), *Juvenile hormone esterase* (Lnig_g08195), *Insulin* (Lnig_g17128), *Protein doublesex* (Lnig_g04508), *Protein henna* (Lnig_g13064), *Tret1a* (Lnig_g01559), *Tret1b* (Lnig_g11948) and *Tret1‐2 homologue* (Lnig_g10144). The other genes of interest did not differ between queens with workers and queens with separated workers (Figure S4, Table S4), indicating that their expression changes differed from the behavioural response (Majidifar et al. 2024).

### 3.5 Effect of the Presence of Larvae on the Expression of the Genes of Interest (Experiment C)

The experimental addition of workers affects the brood care behaviour of queens (Majidifar et al. 2024) and is thus confounded with whether or not queens interact with larvae: queens without workers have many interactions with larvae, while queens with workers have few or no interactions with larvae. Thus, our differential expression analysis may have captured expression changes that were the consequence of interacting with larvae rather than the response to the experimental manipulation of the presence of workers. To verify that changes in the expression of the genes of interest were not the mere consequence of interacting with larvae, we compared brain gene expression between founding queens that were kept with six larvae or without larvae. None of those queens were provided with workers, thus the queens with larvae expressed brood care and interacted with larvae, and the queens without larvae did not.

We predicted that if the effect of the presence of workers on the expression of the genes of interest was the mere consequence of interacting with larvae, we would detect expression differences between queens with and without larvae in a specific direction: genes overexpressed in queens kept with workers (that had few or no interactions with brood) would be overexpressed in queens without larvae, and genes overexpressed in queens kept without workers (that had many interactions with brood) would be overexpressed in queens with larvae. We found that nine of the 13 genes of interest did not differ between queens with and without larvae (Figure 3, Figure S5, Table S5): *Insulin* (Insulin‐like_Lnig_g17128), *Juvenile hormone esterase* (Lnig_g08195), *Esterase E4* (Lnig_g15862), *Protein doublesex* (Lnig_g04508), *Protein henna* (Lnig_g13064), *Prohormone‐2* (Lnig_g06899), *GR81* (gLnigGR81), *Tret1a* (Lnig_g01559) and *Tret1b* (Lnig_g11948). The other four genes of interest were affected by the presence of larvae, but in the opposite direction to what would be expected if the effect of the presence of workers was explained by an effect of interacting with larvae: *Kr‐h1* (Lnig_g01448) and *PDK1* (Lnig_g11740) were more expressed with larvae (Figure 3, Figure S5, Table S5), but less expressed in absence of workers (Figure 2), while *Pheromone‐binding protein GP9* (gLnigOBP1) and *Tret1‐2 homologue* (Lnig_g10144) were more expressed without larvae (Figure 3, Figure S5, Table S5), but less expressed in presence of workers (Figure 2).

## 4 Discussion

Ant queens transition from expressing behavioural pluripotency during colony foundation to becoming specialised in egg production after worker emergence. Although the behavioural response of queens to changes in the social environment has been well characterised (Cassill 2002; Majidifar et al. 2024; Woodard et al. 2013), there is limited understanding of the molecular mechanisms that translate the presence of workers into queen specialisation. To address this question, we produced and analysed brain RNA‐seq data from 93 age‐matched *L. niger* queens in multiple, experimentally manipulated social environments (without workers, with workers, formerly with workers, with workers separated by a wire mesh, with larvae and without larvae). The comparison of queens with and without workers revealed 123 differentially expressed genes (DEGs), including 13 genes of interest with reported roles in regulating insect behaviour via their expression in the brain. Of these genes of interest, four showed expression patterns that mirrored the behavioural response to both the removal of workers (Experiment A) and the addition of separated workers (Experiment B): *Kr‐h1*, *Juvenile hormone esterase*, *Insulin* and *Tret1‐2 homologue*.

The enrichment analysis of the list of DEGs identified three categories of processes and functions that were associated with changes in the social environment and/or the behavioural response of queens. The first category consisted of GO terms related to energy reallocation, such as triglyceride synthesis and catabolism, glycogen synthesis, glucose binding and trehalose transport. It is important to note that none of the queens were provided with food, irrespective of worker presence, thus these patterns of energy reallocation in the brain did not merely stem from nutritional differences. Rather, they likely reflected variation in brain activity, as the functioning of neurons (e.g., production and integration of signalling molecules, generation of action potentials) requires energy metabolism (Mergenthaler et al. 2013). We propose that founding queens kept without workers and that provided care to the brood mobilised energy via triglyceride catabolism and carbohydrate transport and binding to meet the high‐energy demands of expressing brood care behaviour. In line with this hypothesis, behavioural variation in honey bee workers was associated with differences in brain energy metabolism (Rittschof et al. 2015; Rittschof and Schirmeier 2018), including in response to social interactions (Rittschof et al. 2018). Conversely, the experimental addition of workers to founding queens likely induced a shift toward brain energy conservation and storage, as indicated by changes in triglyceride and glycogen biosynthesis.

The second functional category grouped GO terms involved in membrane remodelling and neuronal plasticity, including phosphatidylserine and phosphatidylglycerol metabolic processes, and high‐density lipoprotein particle remodelling. These enrichments suggest that the queen brain may be undergoing structural modifications in response to variation of the social environment and/or associated with the behavioural changes. This interpretation is supported by previous reports of neuroanatomical plasticity in other social insect species. First, the social environment and dominance interactions experienced by facultatively social bees resulted in variation in the relative volume of the mushroom bodies (Rehan et al. 2015; Smith et al. 2010). Second, the volume of this part of the brain correlated with behavioural variation in honey bee and ant workers (Withers et al. 1993; Valadares et al. 2026) and with social aggression in a paper wasp (Molina and O'Donnell 2008). Interestingly, workers of the ant *Camponotus floridanus* experimentally kept with and without larvae—and thus expressing brood care or not—differed in the relative volumes of the antennal lobes and the mushroom bodies (Gronenberg et al. 1996). Such variation in brain structure also occurred after performing foraging tasks in several species of bees (Jaumann et al. 2022; Withers et al. 1993; Fahrbach et al. 1997) and was associated with age in both honey bees (Fahrbach et al. 1995) and wasps (Mattens et al. 2026). Third, mated queens of the ants *Messor pergandei* , *Pogonomyrmex rugosus* and *Monomorium pharaonis* showed reduced volume of the brain and/or specific brain parts when compared to unmated gynes, as well as behavioural differences in mobility, attraction to light or digging behaviour (Nagel et al. 2020; Julian and Gronenberg 2002). Such reduction was also documented for established mated queens (with workers and brood) collected several months after mating compared to younger, newly mated queens (without workers nor larvae) collected 5 days after the nuptial flight (Julian and Gronenberg 2002). Similarly, the brain volume was reduced in established termite queens and kings compared to dispersing, winged individuals (Castillo and Sun 2026). Finally, in the ant *H. saltator* , reproductive workers (gamergates) showed a strong reduction in total brain volume compared to foragers (Penick et al. 2021). This reduction was found to be reversible, as the experimental reversal of gamergates to becoming non‐reproductive workers was accompanied with an increase in brain volume (Penick et al. 2021). Interestingly, one study that reported brain structural changes associated with the mating status of ant queens also found that these changes were reflected in the brain transcriptomic analyses, as differentially expressed genes between mated queens and unmated gynes were involved in neuronal activity (Nagel et al. 2020). However, whether our finding that *L. niger* queens with and without workers differed in the expression of genes involved in membrane remodelling and neuronal plasticity actually reflects that brain structural changes are implicated in their behavioural specialisation remains to be investigated.

The third functional category contained GO terms associated with transmembrane transport and the cellular response to neuropeptides, which likely reflect variation in neuronal homeostasis and signalling between queens with and without workers. Finding enrichment for terms related to transmembrane transport indicates that the experimental manipulation of workers affected the release, transport and/or re‐uptake of neurotransmitters, consistent with the implication of neurotransmission in generating behavioural variation (Barbero and Casacci 2025). More specifically, the enriched term cellular response to peptides suggests the implication of neuropeptides, which regulate various aspects of division of labour and behavioural specialisation in ants. First, the production and release of the neuropeptide insulin‐like peptide 2 controls inter‐individual variation in reproductive activity (Libbrecht et al. 2018; Qiu et al. 2018; Chandra et al. 2018). Second, the gene coding for the neuropeptide neuroparsin A shows differential brain expression associated with reproductive and behavioural variation: between unmated gynes and mated queens (Nagel et al. 2020), between unmated gynes and workers (Qiu et al. 2018), and between reproductive and non‐reproductive clonal ants (Nagel et al. 2020; Libbrecht et al. 2018). Third, the neuropeptide corazonin controls the behavioural changes associated with the transitions from non‐reproductive workers to gamergates in *H. saltator* (Gospocic et al. 2017). Finally, the neuropeptide inotocin modulates age‐associated behavioural variation in the clonal raider ant *O. biroi* (Fetter‐Pruneda et al. 2021).

In addition to conducting enrichment analyses to identify functional categories in the list of DEGs, we manually curated the list to identify 13 genes of interest, which were reported to be expressed in insect brains and have putative functions in regulating behaviour (Table 2). These genes of interest included genes involved in JH and insulin signalling pathways, chemoreception, neurotransmitter production and trehalose transport. Further analyses revealed that four of the 13 genes of interest fully mirrored the behavioural responses to various manipulations of the social environment (Table 3). First, we found evidence that their expression changes in response to the addition of workers were reversible (Experiment A, Figure S2), consistent with the reversibility of the queen behavioural specialisation upon worker removal (Majidifar et al. 2024). Second, the expression of those genes did not change in response to the mere presence of workers' cues (Experiment B, Figure S4), which aligns to the absence of queen behavioural changes in the same experimental conditions (Majidifar et al. 2024). Third, we found no evidence that the expression differences between queens with and without workers were driven by queens interacting more with larvae in the condition without workers (Experiment C, Figure S5). The four genes of interest that fulfilled those three criteria (*Kr‐h1*, *Juvenile hormone esterase*, *Insulin*, *Tret1‐2 homologue*) are candidate genes for the molecular regulation of the social control of queen specialisation in ants.

**TABLE 3:** Classification of the genes of interest depending on their brain expression patterns.

Gene annotation	Experiment A: Evidence that gene expression changes in response to worker presence are reversible	Experiment B: Evidence that antennations with workers through a wire mesh is not sufficient to produce gene expression changes	Experiment C: Evidence that interactions with larvae actually explain the apparent effect of workers on gene expression
***Kr‐h1***	**YES**	**YES**	**NO**
*PDK1*	NO	YES	NO
***Insulin***	**YES**	**YES**	**NO**
***Juvenile hormone esterase***	**YES**	**YES**	**NO**
*Esterase E4*	NO	NO	NO
*Protein doublesex*	NO	YES	NO
*Protein henna*	NO	YES	NO
*Prohormone‐2*	YES	NO	NO
*Pheromone‐binding protein Gp‐9*	YES	NO	NO
*GR81*	NO	NO	NO
*Tret1a*	NO	YES	NO
*Tret1b*	NO	YES	NO
***Tret1‐2 homologue***	**YES**	**YES**	**NO**

*Note:* Our analyses identified four candidate genes (in bold) that mirrored the behavioural response to multiple experimental manipulations of the presence of workers and for which we found no evidence that the expression changes were a mere consequence of interacting with larvae.

The first candidate gene, *Krüppel‐homologue 1* (*Kr‐h1*), codes for a JH‐responsive transcription factor and is upregulated in the brain of queens that reduced their brood care behaviour in response to the experimental addition of workers. The expression of *Kr‐h1* in the brain is involved in regulating other behavioural and reproductive transitions in social insects. In bees, *Kr‐h1* expression correlates with the age‐associated behavioural transitions of workers (Shpigler et al. 2010; Fussnecker and Grozinger 2008), and with the reproductive activation of workers upon queen removal (Shpigler et al. 2010), likely via its downregulation in response to queen mandibular pheromones (Grozinger and Robinson 2007). In the ant *H. saltator* , *Kr‐h1* controls the behavioural transition from workers that express non‐reproductive behaviours to reproductively active gamergates (Opachaloemphan et al. 2023; Gospocic et al. 2021). Specifically, *Kr‐h1* brain expression was proposed to repress the expression of behaviours that should not be expressed in a particular social context by regulating downstream transcriptomic changes in response to the socially responsive JH pathway (Gospocic et al. 2021). Our findings that *Kr‐h1* brain expression in *L. niger* queens correlates with their social environment and behavioural specialisation supports a similar role in translating the presence of workers into reduced brood care, possibly via JH‐dependent regulation of downstream gene expression.

This hypothesis is supported by the finding that two other candidate genes belong to the JH and insulin signalling pathways. *Juvenile hormone esterase*, upregulated in the brain of queens provided with workers, codes for an enzyme that hydrolyses JH. In social insects, JH is involved in regulating age‐associated behavioural transitions in workers by modulating the expression of *vitellogenin* genes (Hawkings and Tamborindeguy 2024; Kohlmeier et al. 2019, 2018; Amsalem et al. 2014; Corona et al. 2007; Libbrecht et al. 2013), although we found no evidence that the expression of *vitellogenin* genes in the brain of *L. niger* queens was associated with behavioural variation. Experimental treatments with JH or methoprene (a synthetic JH analog) resulted in reduced brood care in workers of the fire ant *S. invicta* (Ren et al. 2023), increased activity, phototaxis and aggression in the leaf‐cutter ant *Acromyrmex octospinosus* (Norman and Hughes 2016), increased foraging in *H. saltator* (Gospocic et al. 2021; Opachaloemphan et al. 2021), age‐dependent effects on foraging and brood care behaviour in workers of the little fire ant *Allomerus auropunctatus* (formerly *Wasmannia auropunctata* (Longino and Branstetter 2026)) (Ortiz‐Alvarado et al. 2021), higher levels of aggressivity in workers of *Polistes* wasps (Giray et al. 2005; Walton et al. 2020), accelerated onset of foraging activities and phototaxis in honey bee workers (Sullivan et al. 2000; Chang et al. 2015; Robinson 1987), and reduced biting behaviour in soldiers of the termite *Reticulitermes aculabialis* (Li et al. 2024). Interestingly, *L. niger* founding queens that received a topical application of methoprene showed reduced brood care levels (Pamminger et al. 2016; Majidifar et al. 2024) while those treated with precocene I (an inhibitor of JH production) increased their brood care behaviour (Majidifar et al. 2024). Assuming that *Juvenile hormone esterase* expression reflects the quantity of JH circulating in the brain, we propose that the experimental addition of workers resulted in elevated brain JH titers that suppressed brood care behaviour.

The other candidate gene associated with the JH and insulin signalling pathways is *insulin*, which codes for an insulin‐like peptide and is overexpressed in response to the experimental addition of workers. Insulin‐like peptides act as hormones, neurotransmitters and growth factors from the insulin signalling pathway, which interacts with JH signalling to regulate fecundity, metabolism and behaviour in insects (Semaniuk et al. 2021; Chandra et al. 2018; Libbrecht et al. 2013). The insulin signalling pathway correlates with vitellogenin production to regulate egg production and lifespan, as well as behavioural variation, in honey bees ( *A. mellifera* ) and the red imported fire ant ( *Solenopsis invicta* ) (Ament et al. 2008; Chen et al. 2023). Interestingly, the gene coding for insulin‐like peptide 2 (*ilp2*) is consistently upregulated in the brain of reproductive ants (queens or gamergates) compared to non‐reproductive workers in many species of ants (Qiu et al. 2018; Chandra et al. 2018), and responds to changes in the social environment independently of nutritional differences (Chandra et al. 2018; Libbrecht et al. 2018), as in this study. Our finding that *insulin* was overexpressed in queens kept with workers—and thus specialised in egg production—may indicate that the insulin signalling pathway linked the presence of workers with changes in reproductive physiology.

The fourth candidate gene, *Tret1‐2 homologue*, encodes a trehalose transporter and is upregulated in the brain of queens provided with workers. Interestingly, trehalose homeostasis is regulated by the insulin signalling pathway (Belgacem and Martin 2006; Xu et al. 2013). Trehalose transporters regulate tissue‐specific uptake of trehalose, the primary circulatory sugar in insects (Saleh et al. 2021; Shukla et al. 2015). In orchid bees ( *Euglossa dilemma* ), *Tret1‐2* brain expression correlated with dominance status, ovary size and reproductive behaviour (Saleh et al. 2021). In *L. niger* workers, the whole‐body expression of *Tret1*, another gene coding for a trehalose transporter, correlated with foraging occurrence, potentially reflecting the high energy demands of this behaviour (Quque et al. 2023). As for the enrichment of GO terms associated with energy metabolism, the fact that queens with and without workers shared the same nutritional conditions indicates that changes in *Tret1‐2 homologue* expression reflect shifts in energy allocation in response to the presence of workers and the associated behavioural specialisation.

While recent work provided robust evidence that the behavioural specialisation of *L. niger* queens is induced and maintained by the presence of workers (Majidifar et al. 2024), the transcriptomic basis of this socially controlled specialisation remained unknown. Here, we applied a well‐replicated, well‐controlled transcriptomic approach combined with targeted social manipulations to identify four candidate genes whose brain expression fully mirrored the behavioural plasticity: *Kr‐h1*, *Juvenile hormone esterase*, *Insulin* and *Tret1‐2 homologue*. Our results indicate that the presence of workers modulates conserved hormonal and metabolic pathways to suppress brood care while promoting behavioural specialisation. Future studies should functionally validate these candidates across social insect lineages and characterise their spatial expression in brain regions governing social behaviour.

## Author Contributions

H.E.L.L. data curation, formal analysis, investigation, methodology, visualisation, writing – original draft, writing – review and editing. M.F.B. data curation, formal analysis, investigation, methodology, visualisation, writing – original draft, writing – review and editing. T.J.C. funding acquisition, methodology, resources, supervision, writing – review and editing. R.L. conceptualisation, data curation, formal analysis, funding acquisition, investigation, methodology, project administration, resources, supervision, validation, visualisation, writing – original draft, writing – review and editing.

## Funding

This project was funded by the Agence Nationale de la Recherche (ANR, French Research Agency) ANR‐23‐CE02‐0004 (project ANTOGENY) (R.L.) and the Deutsche Forschungsgemeinschaft (DFG, German Research Foundation) GRK2526/1‐407023052 (T.J.C. and R.L.).

## Ethics Statement

The research project was conducted on invertebrate species that do not require institutional ethical review or approval. All the research complied with applicable laws on sampling from natural populations and animal experimentation, and the experiments were performed in accordance with directive 2010/63/EU of the European Union.

## Conflicts of Interest

The authors declare no conflicts of interest.

## Supporting information


**Figure S1:** Volcano plot showing the genes of interest among the differentially expressed genes (DEG). Coloured dots and annotations represent the 13 genes of interest among the 123 DEG between queens without (*n* = 24) and with (*n* = 24) workers (adjusted *p* < 0.05; above the red dashed line). Green dots and annotations are genes of interest that were overexpressed in the brain of queens without workers. Blue dots and annotations are genes that were overexpressed in the brain of queens that were experimentally provided with workers.
**Figure S2:** Effect of worker removal on queen brain expression of the genes of interests. Some genes of interest mirrored the behavioural response to worker removal in that they showed a reversible pattern of brain gene expression (gene ID in black), while other genes of interest were not affected by worker removal (gene ID in grey). Coloured points and error bars represent mean ± SE of normalised read counts for queens without workers (green; *n* = 12), queens with workers (blue; *n* = 12) and queens with workers removed (orange; *n* = 12). Letters indicate statistically supported differences between groups. Grey dots represent individual data points.
**Figure S3:** We conducted video analyses (focus animal sampling) to verify that the queens could detect the presence of workers that were placed on the other side of a wire mesh. We used the videos from Experiment 2.2.12 in Majidifar et al. (2024). Queens were recorded for one hour at three time points (2 h, 6 and 24 h after set‐up, three hours per queen in total). (A) Out of nine analysed queens, eight (88.9%) were observed passing their antennae through or above the wire mesh, and five (55.5%) were observed making antennal contacts with workers through the wire mesh. (B) The five queens that made antennal contacts spent 93.64 ± 74.95 s touching the workers with their antennae (min = 11.7 s; max = 164.01 s).
**Figure S4:** Effect of workers separated by a wire mesh on queen brain expression of the genes of interests. Some genes of interest mirrored the behavioural response to separated workers in that they showed no change in expression (gene ID in black), while the expression of other genes was affected by the presence of separated workers (gene ID in grey). Coloured points and error bars represent mean ± SE of normalised read counts for queens without workers (green; *n* = 12), queens with workers (blue; *n* = 12) and queens with workers separated by wire mesh (purple; *n* = 9). Letters indicate statistically supported differences between groups. Grey dots represent individual data points.
**Figure S5:** Effect of the presence of larvae on queen brain expression of the genes of interests. We predicted that if the response to the presence of workers of the genes of interest was actually driven by the interactions of queens with larvae, those genes would be overexpressed in both queens with larvae and queens without workers, or in both queens without larvae and queens with workers. None of the genes of interest followed this pattern: either there was no difference in expression between queens with and without larvae, or the genes responded similarly to the presence of larvae and workers. Coloured points and error bars represent the mean ± SE of normalised read counts for queens without larvae (light blue; *n* = 12) and queens with six larvae (dark orange; *n* = 12). Grey dots represent individual data points.

**Table S1:** Differentially expressed genes between brains of same‐age queens kept with and without workers.
**Table S2:** GO term enrichment analysis of the 123 differentially expressed genes.
**Table S3:** Output of the statistical tests comparing the brain expression of the genes of interest among queens with workers, without workers and with workers removed. In bold are genes showing a main effect of the treatment, a difference in expression between queens with workers and with removed workers, and *t* values indicating that the effect was in the same direction for queens without workers and queens with workers removed when compared to queens with workers.
**Table S4:** Output of the statistical tests comparing the brain expression of the genes of interest among queens with workers, without workers and with workers separated by a wire mesh. In bold are genes showing a main effect of the treatment, a difference in expression between queens with workers and with separated workers, and *t* values indicating that the effect was in the same direction for queens without workers and queens with separated workers when compared to queens with workers.
**Table S5:** Output of the statistical tests comparing the brain expression of the genes of interest among queens with and without larvae. In bold are genes that were not affected by the presence of larvae or that were affected by the presence of larvae but in the opposite direction to what would be expected if the effect of the presence of workers was explained by an effect of interacting with larvae.

**Data S1:** Compressed file that contains the code and input data for all the transcriptomic and statistical analyses conducted in this study.

## Data Availability

The code and input data for all analyses are provided in Data S1. The raw RNA‐seq sequences used in this study are publicly available from the NCBI BioProject PRJNA1548310.
