# Supplementary material for: Brain Transcriptomic Changes Associated With the Socially Regulated Behavioural Specialisation of Ant Queens
Source: Mol Ecol. 2026 Nov 1;35(21):e70614. doi: 10.1111/mec.70614 (PMC13724878; doi:10.1111/mec.70614)
Supplement: Supplementary file 1 [file MEC-35-e70614-s001.pdf]

Brain transcriptomic changes associated with the socially regulated behavioral specialization of ant queens

Hugo Le Lay[1]*, Maximilian F. Bolder[2]*, Thomas J. Colgan[2], Romain Libbrecht[1,2]

[1]Insect Biology Research Institute, UMR 7261, CNRS, University of Tours, Tours, France
[2]Institute of Organismic and Molecular Evolution, Johannes Gutenberg University, Mainz, Germany
*contributed equally

Correspondence: romain.libbrecht@cnrs.fr

Supplementary Figures

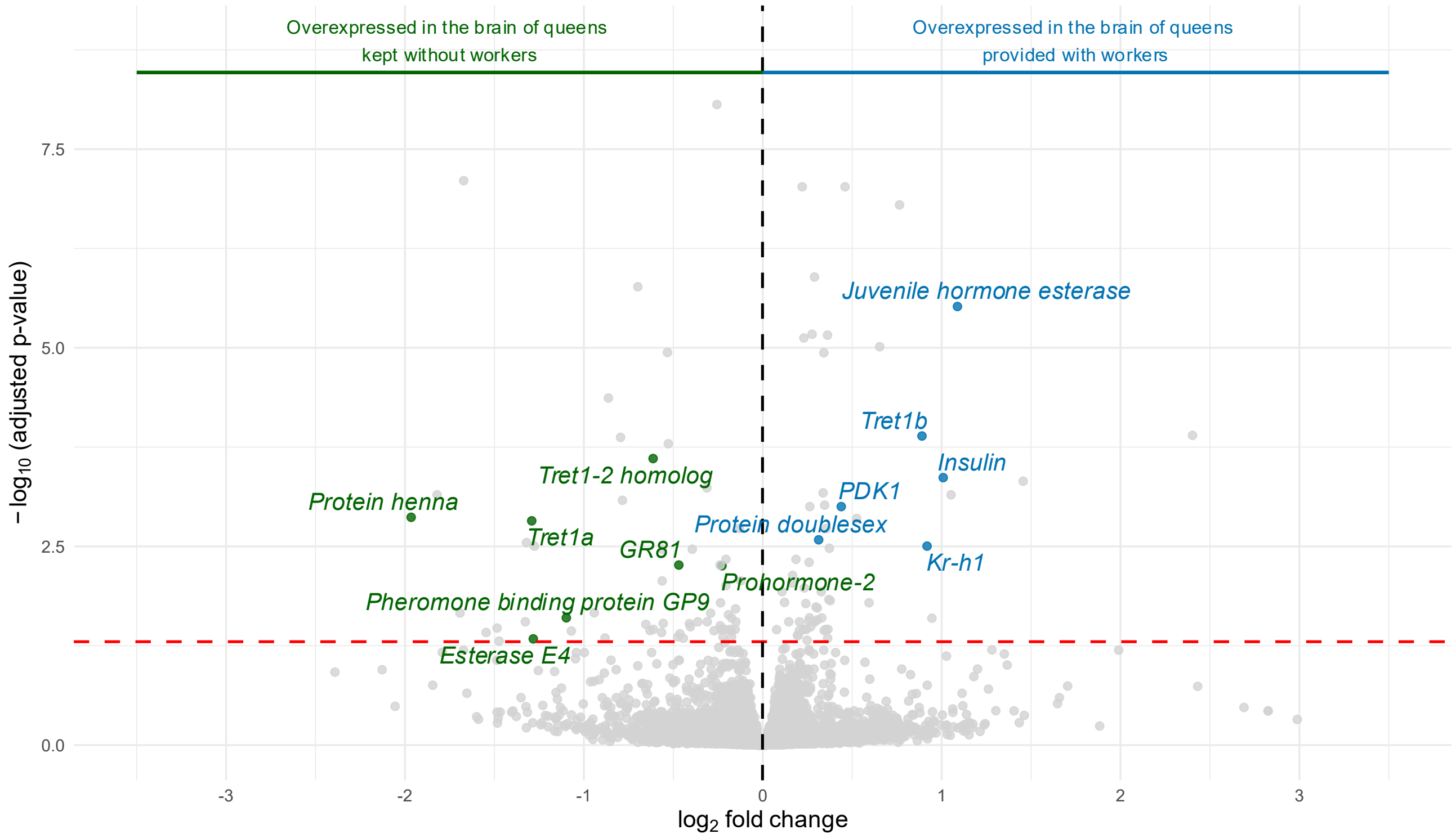

Supplementary Figure 1: Volcano plot showing the genes of interest among the differentially expressed genes (DEG). Colored dots and annotations represent the 13 genes of interest among the 123 DEG between queens without (n = 24) and with (n = 24) workers (adjusted $p < 0.05$; above the red dashed line). Green dots and annotations are genes of interest that were overexpressed in the brain of queens without workers. Blue dots and annotations are genes that were overexpressed in the brain of queens that were experimentally provided with workers.

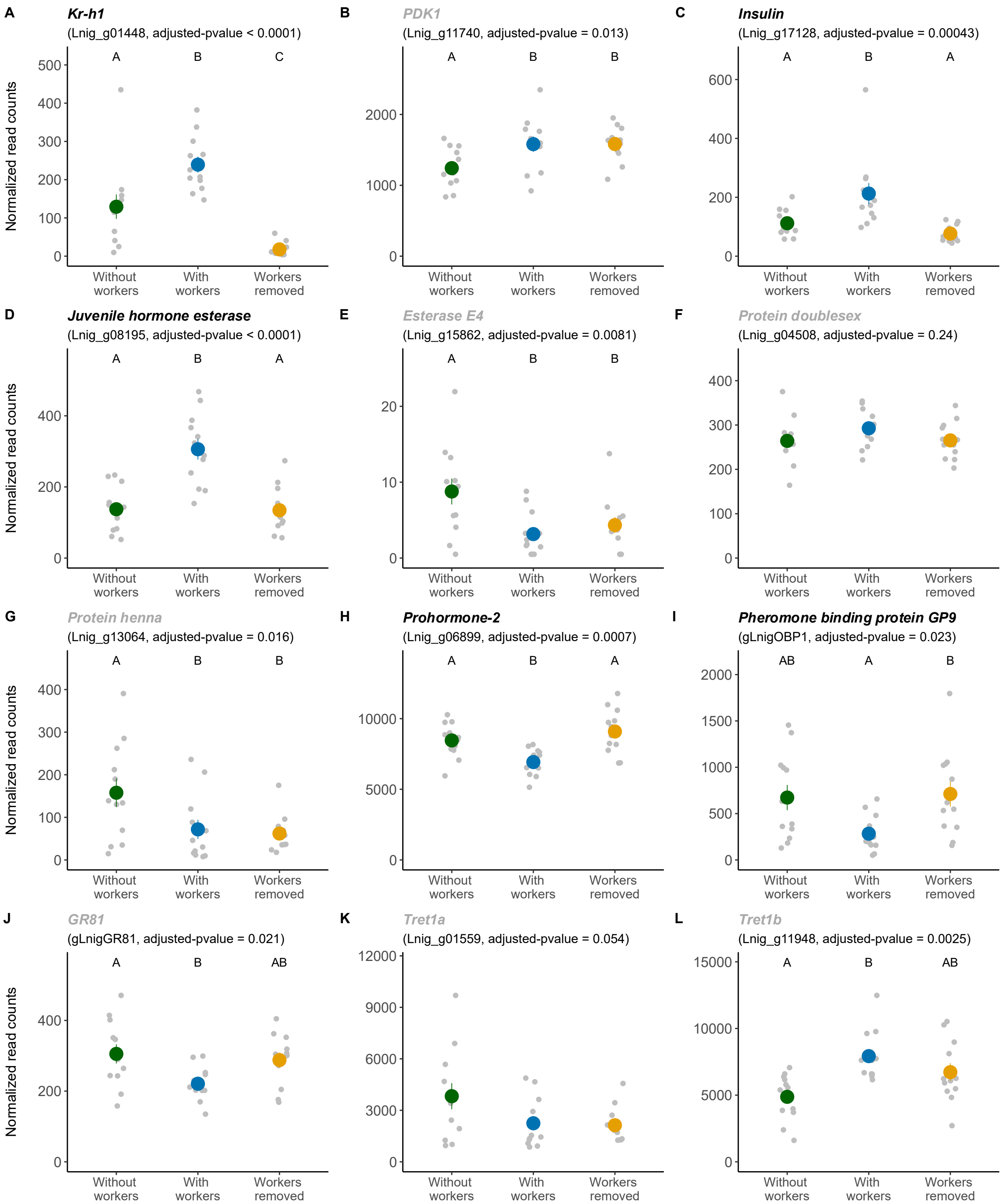

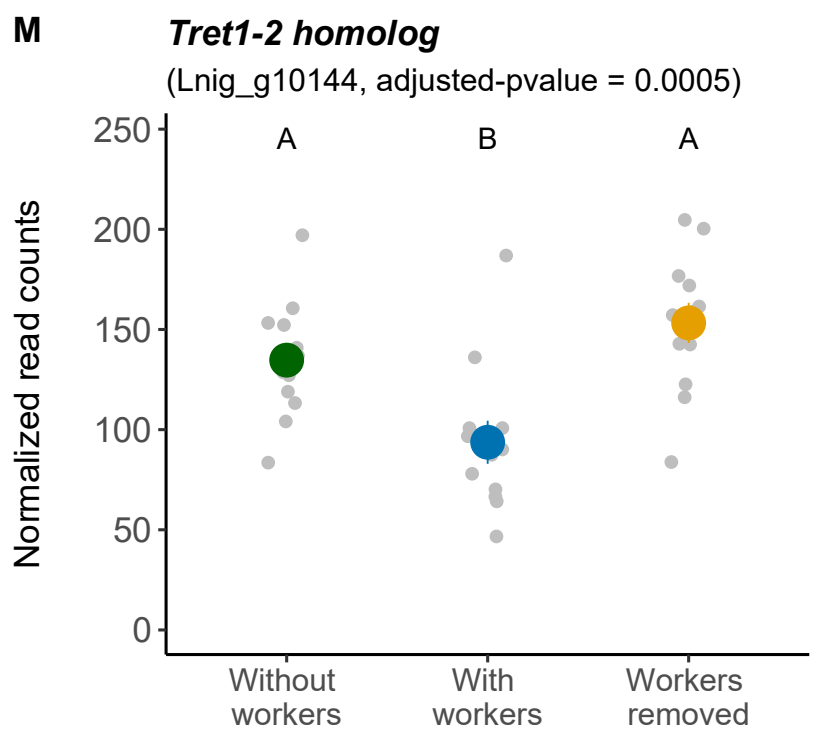

Supplementary Figure 2. Effect of worker removal on queen brain expression of the genes of interests. Some genes of interest mirrored the behavioral response to worker removal in that they showed a reversible pattern of brain gene expression (gene ID in black), while other genes of interest were not affected by worker removal (gene ID in grey). Colored points and error bars represent mean ± se of normalized read counts for queens without workers (green; n = 12), queens with workers (blue; n = 12) and queens with workers removed (orange; n = 12). Letters indicate statistically supported differences between groups. Grey dots represent individual data points.

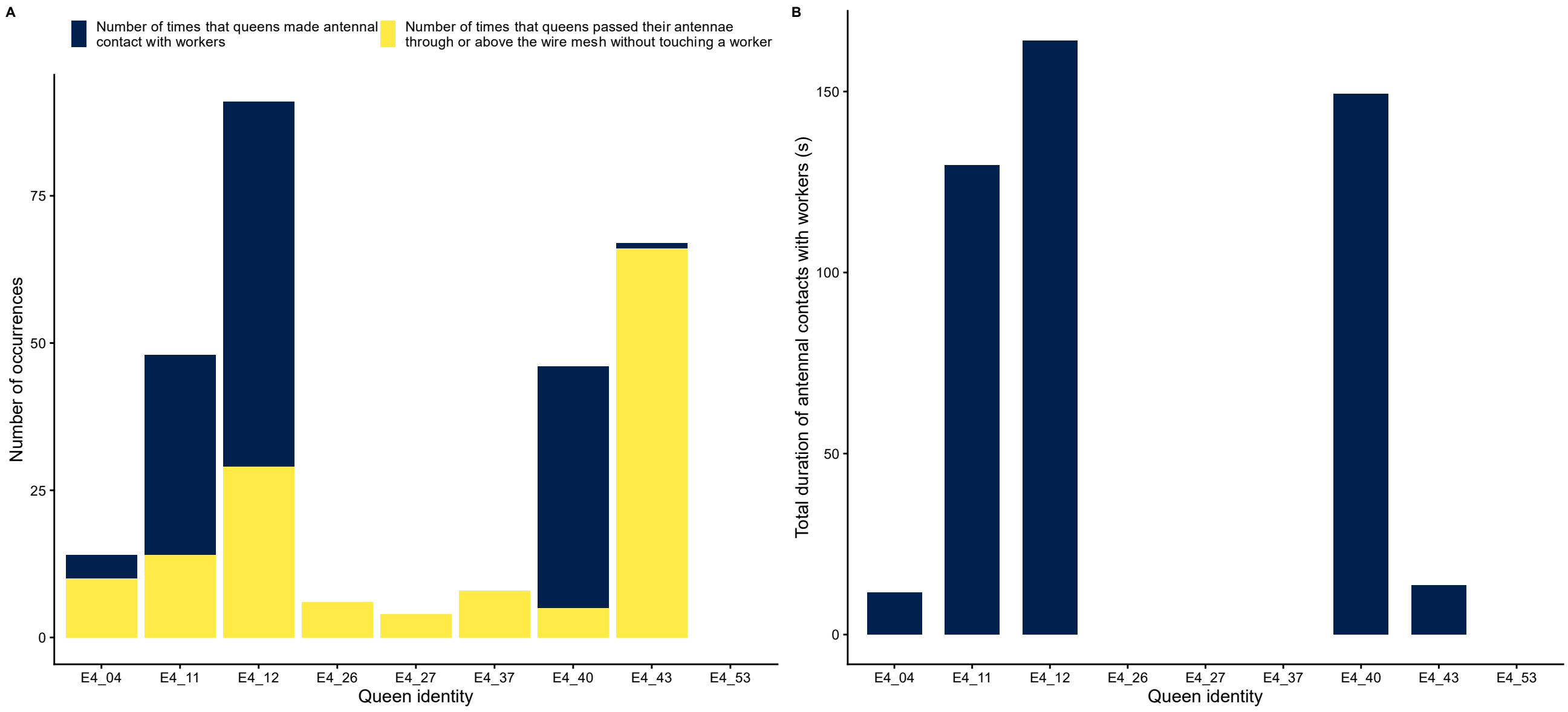

Supplementary Figure 3: We conducted video analyses (focus animal sampling) to verify that the queens could detect the presence of workers that were placed on the other side of a wire mesh. We used the videos from experiment 2.2.12 in Majidifar et al. (2024). Queens were recorded for one hour at three time points (2h, 6h and 24h after set-up, three hours per queen in total). (A) Out of nine analyzed queens, eight (88.9%) were observed passing their antennae through or above the wire mesh, and five (55.5%) were observed making antennal contacts with workers through the wire mesh. (B) The five queens that made antennal contacts spent 93.64 ± 74.95 s touching the workers with their antennae (min = 11.7 s; max = 164.01 s).

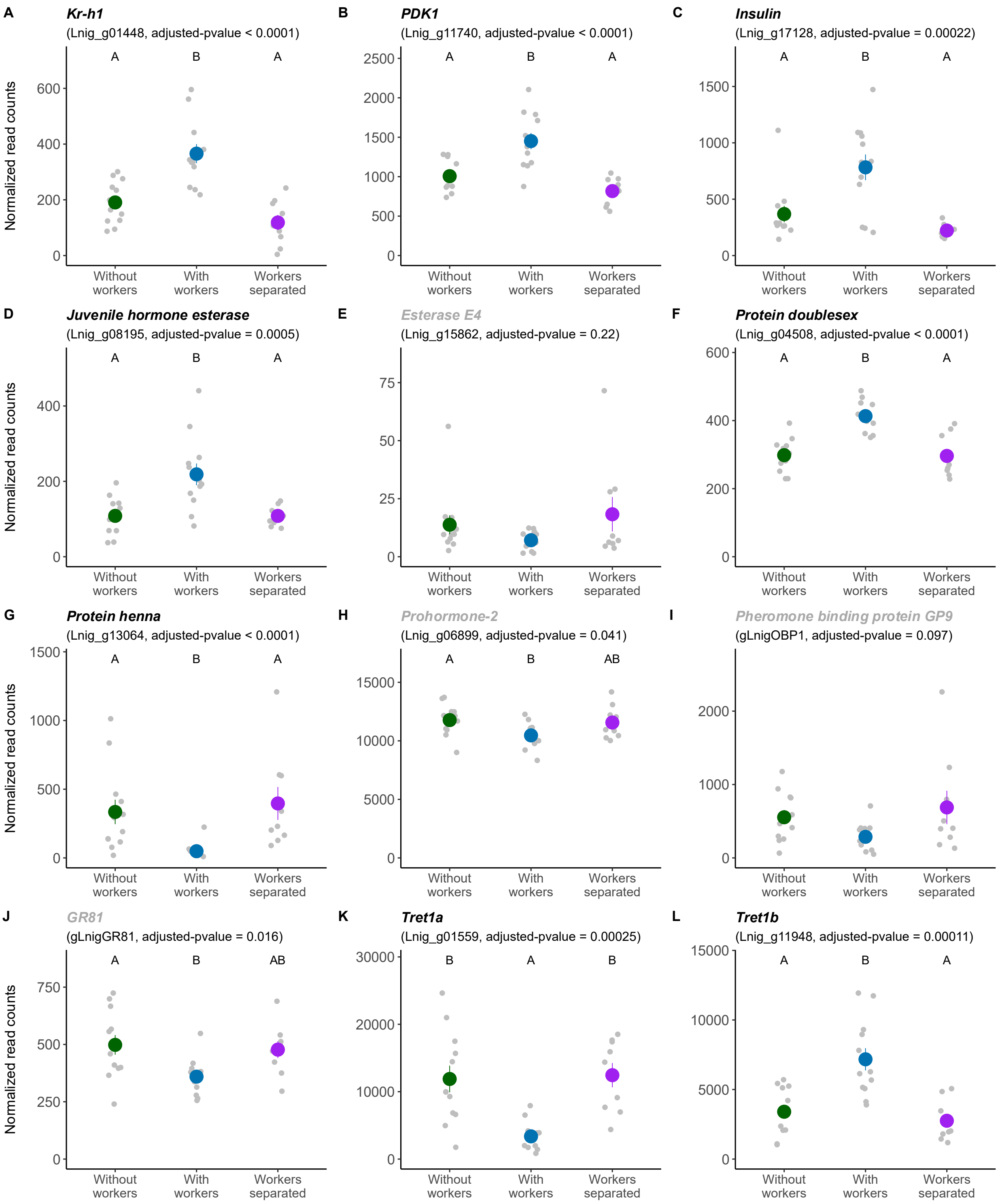

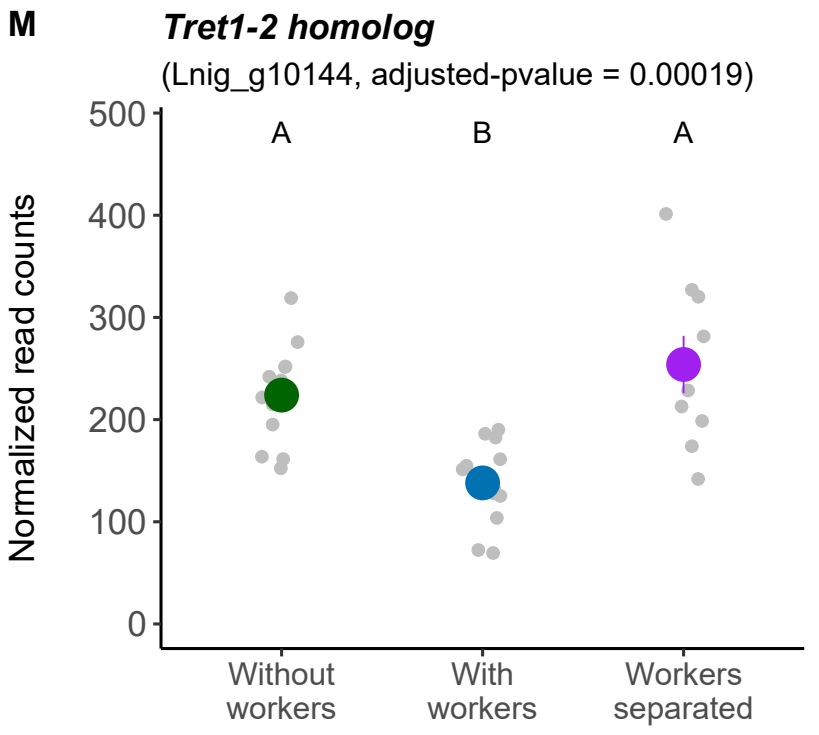

Supplementary Figure 4. Effect of workers separated by a wire mesh on queen brain expression of the genes of interests. Some genes of interest mirrored the behavioral response to separated workers in that they showed no change in expression (gene ID in black), while the expression of other genes was affected by the presence of separated workers (gene ID in grey). Colored points and error bars represent mean ± se of normalized read counts for queens without workers (green; n = 12), queens with workers (blue; n = 12), and queens with workers separated by wire mesh (purple; n = 9). Letters indicate statistically supported differences between groups. Grey dots represent individual data points.

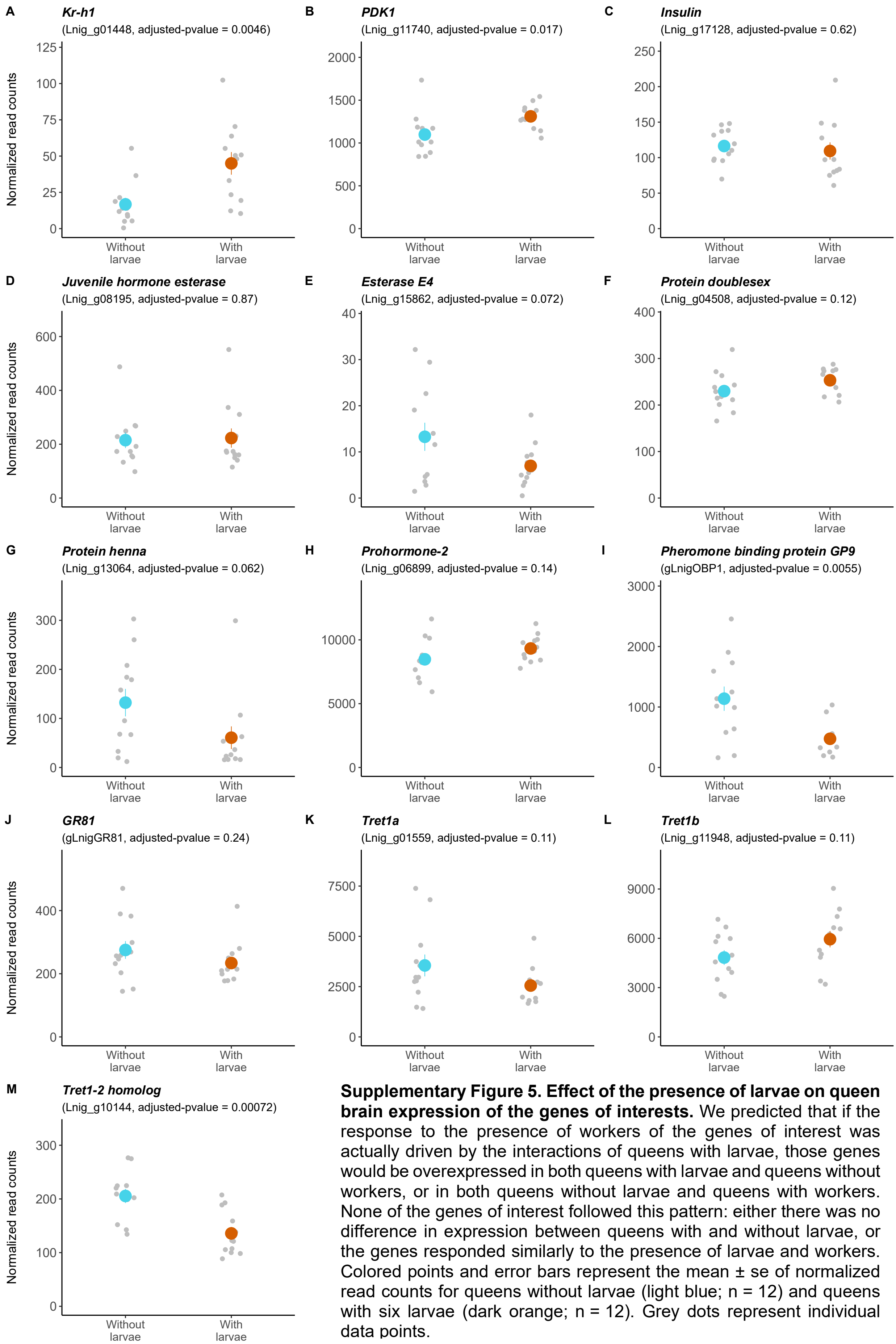

Supplementary Figure 5. Effect of the presence of larvae on queen brain expression of the genes of interests. We predicted that if the response to the presence of workers of the genes of interest was actually driven by the interactions of queens with larvae, those genes would be overexpressed in both queens with larvae and queens without workers, or in both queens without larvae and queens with workers. None of the genes of interest followed this pattern: either there was no difference in expression between queens with and without larvae, or the genes responded similarly to the presence of larvae and workers. Colored points and error bars represent the mean ± se of normalized read counts for queens without larvae (light blue; n = 12) and queens with six larvae (dark orange; n = 12). Grey dots represent individual data points.
